# Evidencing a place for the hippocampus within the core scene processing network

**DOI:** 10.1002/hbm.23275

**Published:** 2016-10-06

**Authors:** C.J. Hodgetts, J.P. Shine, A.D. Lawrence, P.E. Downing, K.S. Graham

**Affiliations:** ^1^ Wales Institute of Cognitive Neuroscience School of Psychology, Cardiff University Cardiff United Kingdom; ^2^ Cardiff University Brain Research Imaging Centre (CUBRIC) School of Psychology, Cardiff University Cardiff United Kingdom; ^3^ Wales Institute of Cognitive Neuroscience School of Psychology, Bangor University Bangor United Kingdom

**Keywords:** functional localiser, fMRI, probabilistic overlap, medial temporal lobe, individual‐level, region‐of‐interest

## Abstract

Functional neuroimaging studies have identified several “core” brain regions that are preferentially activated by scene stimuli, namely posterior parahippocampal gyrus (PHG), retrosplenial cortex (RSC), and transverse occipital sulcus (TOS). The hippocampus (HC), too, is thought to play a key role in scene processing, although no study has yet investigated scene‐sensitivity in the HC relative to these other “core” regions. Here, we characterised the frequency and consistency of individual scene‐preferential responses within these regions by analysing a large dataset (*n* = 51) in which participants performed a one‐back working memory task for scenes, objects, and scrambled objects. An unbiased approach was adopted by applying independently‐defined anatomical ROIs to individual‐level functional data across different voxel‐wise thresholds and spatial filters. It was found that the majority of subjects had preferential scene clusters in PHG (max = 100% of participants), RSC (max = 76%), and TOS (max = 94%). A comparable number of individuals also possessed significant scene‐related clusters within their individually defined HC ROIs (max = 88%), evidencing a HC contribution to scene processing. While probabilistic overlap maps of individual clusters showed that overlap “peaks” were close to those identified in group‐level analyses (particularly for TOS and HC), inter‐individual consistency varied across regions and statistical thresholds. The inter‐regional and inter‐individual variability revealed by these analyses has implications for how scene‐sensitive cortex is localised and interrogated in functional neuroimaging studies, particularly in medial temporal lobe regions, such as the HC. *Hum Brain Mapp 37:3779–3794, 2016*. © **2016 Wiley Periodicals, Inc**.

## INTRODUCTION

The ability to accurately perceive and navigate one's environment is a fundamental, ecologically relevant, cognitive function [O'Keefe and Nadel, 1978]. As such, it is unsurprising that functional neuroimaging techniques have revealed a putative “core” scene processing network in the human brain that responds strongly when viewing navigationally relevant stimuli (e.g., scenes) versus other visual categories. This core network is thought to include posterior parahippocampal gyrus [PHG; Aguirre et al., 1998a; Epstein et al., 2003; Epstein and Kanwisher, 1998], retrosplenial cortex [RSC; Auger et al., 2012; Epstein et al., 2007; Vann et al., 2009] and the transverse occipital sulcus [TOS; Dilks et al., 2013; Ganaden et al., 2013; He et al., 2013; Mullin and Steeves, 2011; Nasr et al., 2011]. As seen in the neural processing of other visual categories [Taylor and Downing, 2011], these regions appear to support distinct but complementary aspects of scene processing, and are differentially modulated by changes in viewpoint [Epstein et al., 2003, 2007; Park and Chun, 2009], spatial layout [Harel et al., 2013; Park et al., 2015], and lower‐level spatial features [Kravitz et al., 2011a; Nasr et al., 2014]. Importantly, while functional differences exist between these different regions, they all share the critical property of showing a preferential response to scenes/places.

The hippocampus (HC) is also considered to play an important role in scene processing [Bird and Burgess, 2008; Lee et al., 2012; O'Keefe and Nadel, 1978]. Beyond the seminal work in both rats and non‐human primates—which identified HC cells attuned to allocentric location [O'Keefe and Nadel, 1978] and spatial view [Rolls, 1999]—recent models of human medial temporal lobe (MTL) function highlight the HC as an important structure for scene processing, via a proposed role in representing complex and conjunctive scene stimuli [Graham et al., 2010; Lee et al., 2012; Murray et al., 2007] and/or by contributions to viewpoint‐independent scene construction [Bird and Burgess, 2008; Maguire and Mullally, 2013; Zeidman et al., 2015]. These complex HC scene representations have been shown to support behavioural performance across a range of cognitive domains, including recognition memory [Bird et al., 2008; Taylor et al., 2007], short‐term memory [Hannula et al., 2006; Hartley et al., 2007], working memory [Lee and Rudebeck, 2010a, 2010b; Park et al., 2003], perceptual learning [Mundy et al., 2013], higher‐order perception [Aly et al., 2013; Barense et al., 2005, 2010; Kolarik et al., 2016; Lee et al., 2005b] and scene imagination [Hassabis et al., 2007].

Despite this evidence, few functional magnetic resonance imaging (fMRI) studies have investigated how scene responses in HC compare to other scene‐sensitive areas [although see Köhler et al., 2002; Mundy et al., 2012, for comparisons between HC and posterior PHG]. A fundamental property of this core scene‐processing network is that a strong neural response to scenes (over other visual categories, such as objects or faces) can be identified in the majority of individual subjects [e.g., Bettencourt and Xu, 2013; Downing et al., 2006; Epstein et al., 2003, 2007; Saxe et al., 2006]. An important question, therefore, is whether the HC also responds preferentially and consistently to scenes, as seen in these other “core” scene processing regions. As previous fMRI studies investigating scene perception and memory in the HC have used anatomical or group‐defined regions‐of‐interest (ROIs), rather than functional ROIs defined within individual subjects [Barense et al., 2010; Lee et al., 2008; Mundy et al., 2013], it is currently unclear whether this is the case.

Here, we address this question by conducting a large‐scale analysis of functional localiser data with the aim of providing information about individual‐level activations [i.e., the number of participants with scene‐sensitive voxels in HC relative to other brain areas; see Machielsen and Rombouts, 2000], but also understanding the spatial profile of these individual‐level activations and how it compares to group‐level statistics [e.g., Engell and McCarthy, 2013; Morrison and Downing, 2007; Nieto‐Castañón and Fedorenko, 2012]. While several studies have attempted to characterise the individual‐level consistency of scene‐sensitive brain activations, these studies have either used small sample sizes [Nasr et al., 2011; Spiridon et al., 2006], or focussed on a single scene processing region [e.g., Peelen and Downing, 2005].

For the current study, we concatenated data from a functional localiser task that was used in two separate fMRI studies. The localiser task required participants to complete a one‐back working memory task while viewing blocks of rapidly presented scenes, objects and scrambled objects. Using this large dataset (*n* = 53), we explored individual‐ and group‐level scene responses within our four main ROIs: PHG, RSC, TOS, and the HC. Our main analyses focused on (a) determining the proportion of individuals that activate these different scene processing regions, (b) how consistently these individual‐level scene activations are elicited, and (c) the extent to which patterns in individual‐level data are reflected in group‐averaged statistics.

## MATERIAL AND METHODS

### Participants

The data from 53 individuals were included in this study (23 male; aged = 18–30 years; mean = 22; SD = 3). Of these, 23 were from Watson et al. [2012] and 30 were part of a related unpublished study. If a participant took part in both studies (*n* = 5), only their earliest scan session was used. Based on self‐report, all participants were right‐handed with normal or corrected‐to‐normal vision and had no history of neurological and/or psychiatric disorder. All participants provided informed consent prior to the experiment and were paid £10 per hour. The Cardiff University School of Psychology Research Ethics Committee approved both experiments.

### Stimuli

**Figure 1:**
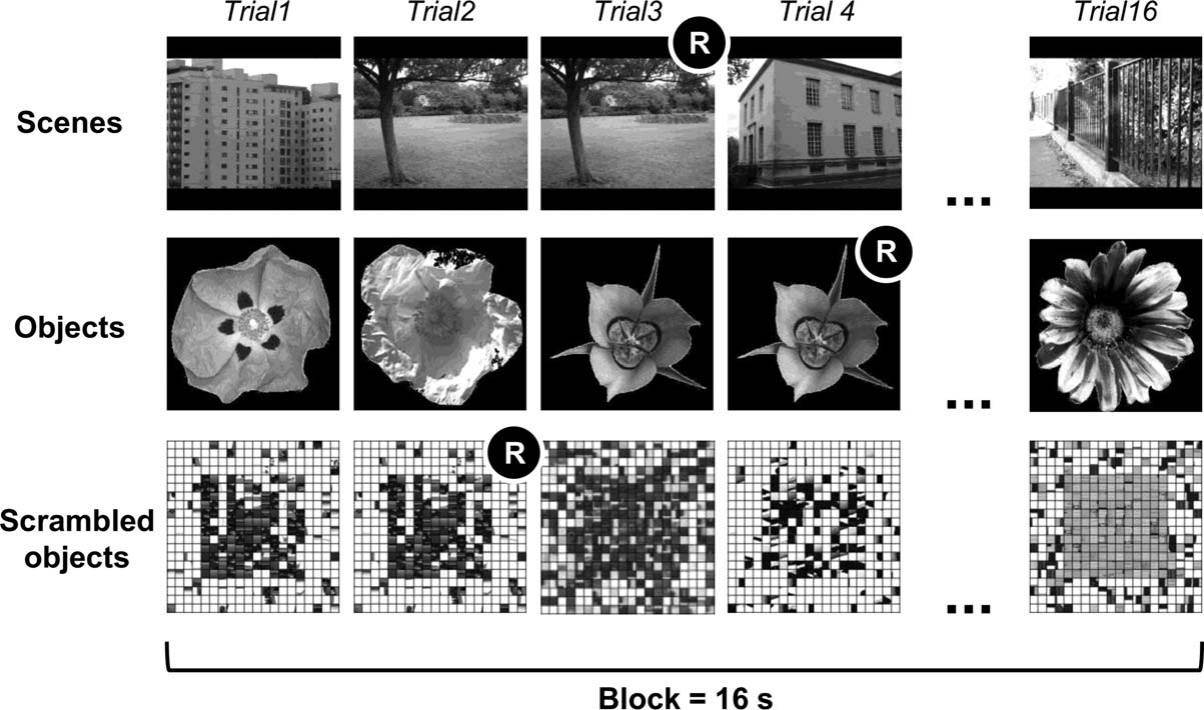
Examples of the three stimulus categories used in the functional localiser task. Each block contained 16 trials with a block duration of 16 seconds. Repeated items (i.e., targets) are marked with an “R.” There were four possible block orders in the task, each presented four times in the task (see “Materials and Methods” section).

Participants were presented with three categories of visual stimuli during the functional localiser task: scenes, objects, and scrambled objects (Fig. 1). Scenes were greyscale real‐world photographs depicting urban areas (i.e., streets, alleys, building exteriors, etc). The objects were greyscale real‐world images of flowers [Mundy et al., 2009]. The scrambled objects were created by taking pictures of familiar objects and overlaying a grid; these individual grid squares were then rearranged concentrically from the middle outward in order maintain the spatial distribution of visual information [Downing et al., 2007]. All stimuli were 400 × 400 pixels.

### Experimental Procedure

The task was programmed and run using the software package Presentation (Neurobehavioral Systems, Albany, CA). The task was projected onto the screen behind the participant using a Canon SX60 LCOS projector system combined with the Navitar SST300 zoom converter lens. Button responses in the scanner were acquired using a right‐hand MR compatible button box. Participants were required to use only their index finger during the task.

The task consisted of 36 experimental blocks (and four fixation blocks), each lasting 16 seconds. During the experimental blocks, greyscale images of scenes, objects, and scrambled objects were presented rapidly (Fig. 1). The task was divided into three distinct block orders: (1) objects, scenes, scrambled objects; (2) scrambled objects, scenes, objects; and (3) scenes, objects, scrambled objects. Each block order was repeated four times resulting in three sets of 12 experimental blocks. Four fixation blocks were interspersed with the category blocks and appeared as blocks 1, 14, 27, and 40. Each stimulus was presented in the centre of a black screen for 200 ms with an ISI of 800 ms resulting in 16 trials per block. Subjects were required to press a button with their index finger when they detected two identical stimuli in immediate succession (i.e., a one‐back task). These stimulus repeats occurred randomly within each block. The mean number of targets across all subjects was 9% of trials (range = 5%–15%) and these were matched across stimulus categories (all 9%). The functional localiser was performed in one run, lasting approximately 11 minutes.

### Imaging Protocol

MRI data were collected using a 3‐Tesla GE HDx MRI (GE Healthcare) system at the Cardiff University Brain Research Imaging Centre (CUBRIC), using an eight‐channel receive‐only head radiofrequency coil. Functional images were acquired using a gradient‐echo, echo‐planar imaging (EPI) sequence. Forty‐five slices were collected per image volume, which were adjusted to ensure whole brain coverage. The fMRI scanning parameters were as follows: repetition time/echo time (TR/TE) 3,000 ms/35 ms; flip angle (FA) 90°; slice thickness 2.4 mm (3.4 × 3.4 × 2.4 mm voxels) with a 1 mm inter‐slice gap; data acquisition matrix GE‐EPI 64 × 64; field of view (FOV) 220 × 220 mm; and ASSET (acceleration factor). The first four volumes were discarded from the localiser run to allow for signal equilibrium. To optimize signal‐to‐noise in MTL regions (i.e., HC), slices were acquired with a 30° oblique axial tilt relative to the anterior‐posterior commissure line [posterior downward; Deichmann et al., 2003]. In order to correct for geometrical distortions and signal loss arising from magnetic field inhomogeneities, high‐resolution fieldmaps were also acquired during the scanning session (TE 7 and 9 ms; TR 20 ms; FA 10°; data acquisition matrix 128 × 64 × 70; FOV 384 × 192 × 210 mm). A high‐resolution anatomical scan was acquired for each participant using a T1‐weighted sequence comprising 178 axial slices (3D FSPGR). Scanning parameters for the structural scan were: FA 20°; data acquisition matrix 256 × 256 × 176; FOV 256 × 256 × 176 mm; and 1 mm isotropic resolution.

### fMRI Preprocessing

The imaging data were preprocessed and analysed using the FMRIB Software Library (FSL, http://www.fmrib.ox.ac.uk/fsl). Participants' T1‐weighted images were stripped of non‐brain tissue using the FSL Brain Extraction Tool [BET; Smith, 2002]. EPI preprocessing and analysis was carried out using the FMRI Expert Analysis Tool (FEAT) Version 5.98. The following pre‐statistics were conducted: motion correction using MCFLIRT [Jenkinson et al., 2002]; brain extraction using BET; high‐pass temporal filtering (Gaussian‐weighted least‐squares straight line fitting, with *σ* = 50 s); and fieldmap unwarping of EPI data using FUGUE [Jenkinson et al., 2002]. Spatial smoothing for subsequent group‐level analyses was carried out with a Gaussian kernel of FWHM 5 mm. The linear registration tool FLIRT [Jenkinson et al., 2002; Jenkinson and Smith, 2001] was used to register participants' EPI data to both their T1‐weighted anatomical scans and to the standard Montreal Neurological Institute (MNI‐152) template image (all coordinates are henceforth reported in MNI space). To achieve greater anatomical specificity at the individual‐level, we also examined the results using a smaller Gaussian smoothing kernel (FWHM 2 mm), and with no additional smoothing (0 mm). While individual‐level consistency is assessed at each level of smoothing (0, 2, and 5 mm), only the 5 mm individual data are combined at the group‐level.

### fMRI Analysis

Following pre‐statistics, each individual participant's four‐dimensional (4D) EPI image was entered into a random‐effects general linear model (GLM). The GLM comprised three predictors: scenes, objects and scrambled objects. Blocks for each condition, corresponding to a regressor duration of 16 s, were convolved with a double gamma haemodynamic response function (HRF) and four main contrasts were implemented: scenes > scrambled objects, scenes > objects, objects > scrambled objects and objects > scenes. Our main contrast for the individual‐level analyses was the direct scene‐selective contrast: scenes > objects [e.g., Marchette et al., 2015; Zeidman et al., 2015]. Parameter estimates reflecting the fit between the voxel‐wise time course and the model were extracted.

For the group‐level analysis, individual parameter estimate images were combined into a mixed‐effects model using FLAME stage 1 [FMRIB's Local Analysis of Mixed Effects; Beckmann et al., 2003; Woolrich et al., 2004]. The resulting whole brain Z statistic images were then thresholded with an initial cluster‐forming threshold of *Z* = 2.3 (i.e., *P* = 0.01). A family‐wise error (FWE) corrected cluster‐extent threshold of *P* < 0.05 was applied based on Gaussian Random Fields (GRF) theory. For ROI‐based inferences, these FWE‐corrected whole brain statistical images were intersected with the anatomical masks outlined below.

In order to (a) avoid biasing the results through selection of an arbitrary threshold, and (b) explore how individual‐level response profiles vary across our putative scene‐sensitive ROIs, we adopted three uncorrected voxel‐wise thresholds for our individual‐level analyses [see Duncan et al., 2009; Engell and McCarthy, 2013]. The individual‐level whole brain *Z* statistic images (across three spatial smoothing filters) for scenes > objects were thresholded with the following criteria: (i) *Z* = 2.3 (*P* = 0.01); (ii) *Z* = 3.1 (*P* = 0.001); and (iii) *Z* = 3.9 (*P* = 0.0001). To evaluate the proportion of subjects with supra‐threshold scene‐selective voxels, we intersected these individual‐level whole brain images (at each threshold and smoothing kernel) with subject‐specific ROIs (described below).

To assess the spatial consistency of individual scene activations within each ROI, we created probabilistic overlap maps by aligning individual activation maps (at each cluster threshold) to the standard template. These standardised individual‐level clusters were then binarised and overlaid to produce probabilistic overlap maps where the value at each non‐zero voxel indicated the number of subjects with supra‐threshold activations at that location [Fedorenko et al., 2010]. To maximise the spatial accuracy of individual‐level HC activations, we aligned only the unsmoothed data.

Percentage signal change values for each participant were derived by extracting the parameter estimates for scenes and objects separately (each relative to the scrambled objects baseline) from each ROI using Featquery in FSL. These were compared both at the group‐level (by averaging across subjects), and at the individual‐level using non‐parametric tests (Cochran's Q) to compare the proportion of subjects with numerically greater responses to scenes relative to objects within each ROI.

### Definition of ROIs

#### Group‐level

**Figure 2:**
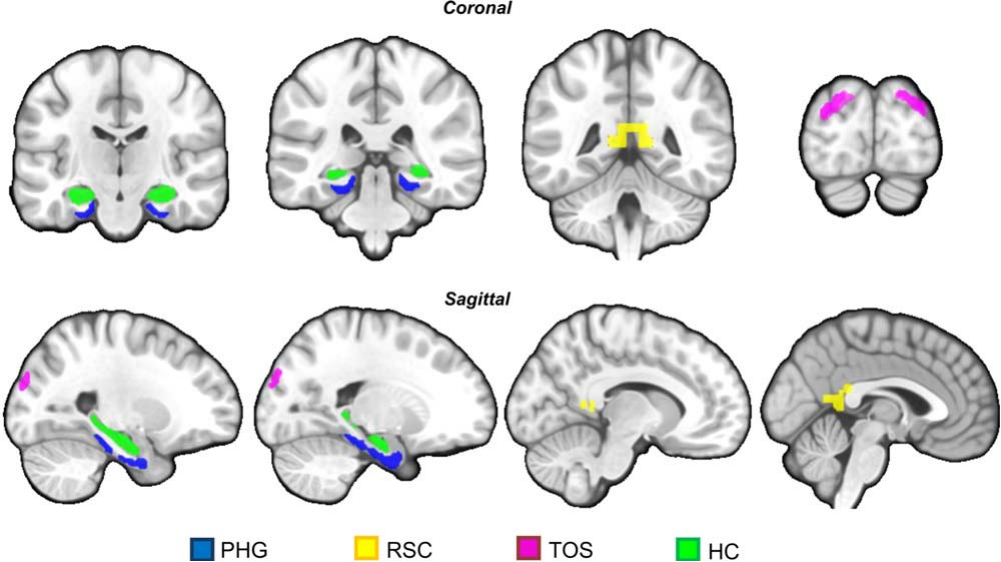
The four anatomical ROIs (PHG, RSC, TOS, and HC) in standard MNI‐152 2 mm template space. The key for the colour‐coded ROIs is shown at the bottom of the figure. [Color figure can be viewed at http://wileyonlinelibrary.com.]

For consistency in the analysis approach across brain regions, and to ensure ROIs were defined independently of functional data [Kriegeskorte et al., 2009], we adopted an anatomical ROI approach.^1^ For group‐level analysis, bilateral ROIs of the PHG and HC were created using probabilistic masks from the Harvard–Oxford cortical and subcortical atlases in FSL, using a probability threshold of 50% (Fig. 2). Both the HC and PHG ROIs incorporated the whole structure. A probabilistic mask for the TOS was created using a probabilistic mask from the ICBM sulcal atlas [Mazziotta et al., 1995] and warped into MNI‐152 space using FLIRT (Fig. 2). Given the anatomical variability of the TOS across individuals (maximum overlap for the TOS in this atlas is 46%), a more liberal probability threshold of 25% was used to define the mask, providing coverage of TOS and partial coverage of the adjacent lateral occipital cortex (LOC) and occipital pole [Nasr et al., 2011]. Using standard anatomical guidelines for identifying sulci [Duvernoy, 1999; Iaria and Petrides, 2007], the placement of the TOS ROI was confirmed on the standard MNI‐152 brain template. The RSC ROI was derived by extracting Brodmann area (BA) 29 from the Talairach atlas [Tan et al., 2013]. There was no spatial overlap between any of the ROIs.

#### Individual‐level

For the individual‐level analyses, we transformed the standard space ROIs for the PHG, RSC and TOS (see above) into individual subject space by inverting the normalisation parameters (the native‐to‐MNI space transformation matrix) provided by FEAT and applying these to the ROIs in standard space. As previous research has reported poor correspondence between the HC defined at the template‐level and individually defined HC [Yassa and Stark, 2009], we used FMRIB's Integrated Registration and Segmentation Tool (FIRST) in FSL to segment HC ROIs on each subject's T1‐weighted structural image. Following segmentation, each individual HC was visually inspected and, if necessary, amended.

## RESULTS

### Behavioural Results

For each participant, hits, misses and false alarms were calculated for scenes, objects and scrambled objects. Only subjects that performed accurately in the one‐back task were included in the subsequent MRI analyses. Two participants were excluded for not registering any correct responses, resulting in a final sample of *n* = 51 for the imaging analyses. Mean hit rate for the remaining participants was 0.77 (SD = 0.12) and the false alarm rate was 0.07 (SD = 0.16). The hit minus false alarm rate for each stimulus category was entered into a one‐way repeated measures ANOVA revealing a main effect of stimulus type (*F*(2, 46) = 22.62, *P* < 0.01). Post‐hoc *t*‐tests (Bonferroni‐corrected) confirmed that performance for scrambled objects (mean = 0.61; SD = 0.20) was significantly poorer than both objects (mean = 0.73; SD = 0.18; *t*(1, 50) = 5.93, *P* < 0.01, d = 1.32) and scenes (mean = 0.75; SD = 0.20; *t*(1, 50) = 4.72, *P* < 0.01, d = 2.3). There was no difference in performance between the scene and object conditions (*P* = 0.47).

### Group‐Level

#### Whole brain analyses

**Figure 3:**
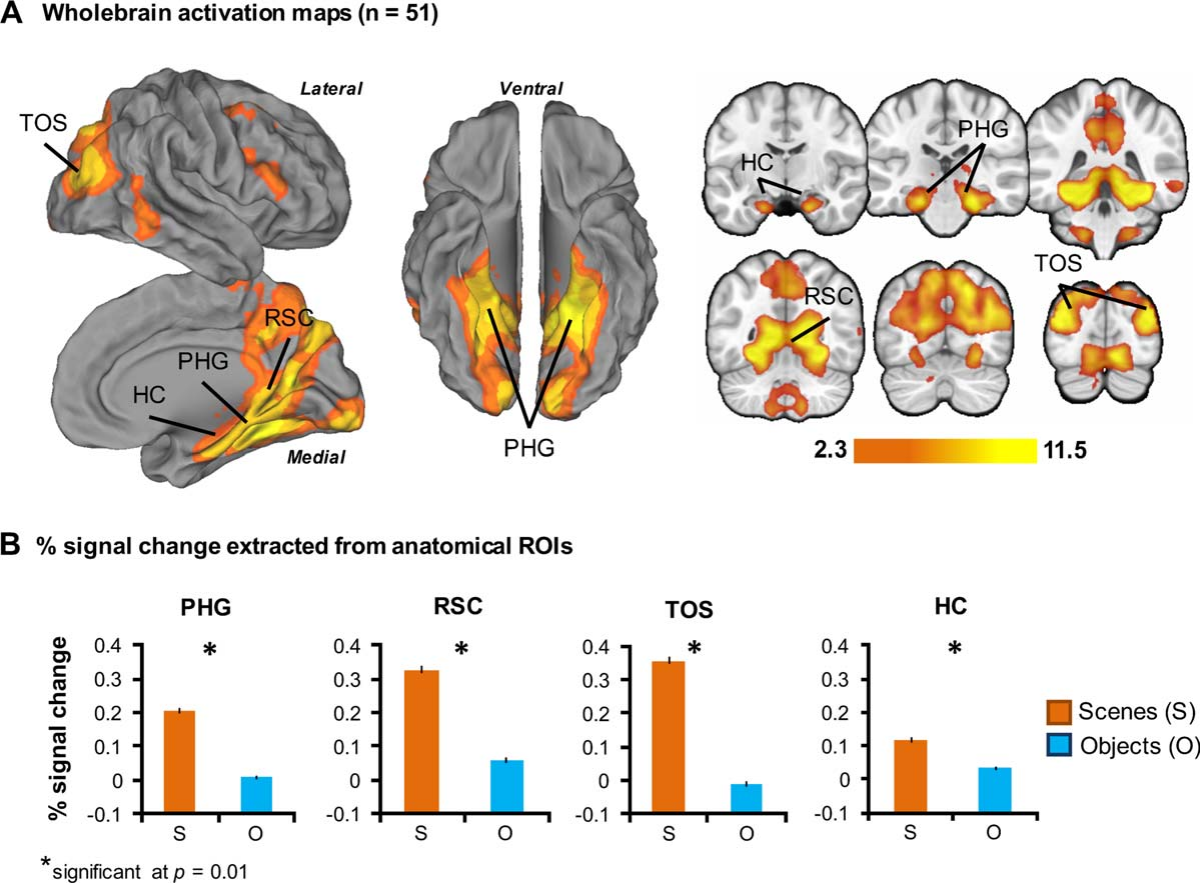
(**A**) Whole brain activity for scenes > objects (*Z* > 2.3, FWE‐corrected *P* < 0.05). Clusters reflecting significantly greater activity for scenes > objects are shown in red‐yellow. Significant scenes > objects activity was found bilaterally in PHG, TOS, RSC, and HC. For visualisation, the activation map was projected onto the PALS surface (PALS‐B12) using Caret (A, left) and to the standard MNI‐152 template (A, right). (**B**) Plots showing mean percent signal change values for scenes and objects (relative to scrambled objects baseline) for each ROI [orange bars = scenes (S); blue bars = objects (O)]. [Color figure can be viewed at http://wileyonlinelibrary.com.]

The random‐effects group‐level (*n* = 51) activation maps are shown in Figure 3A. The scenes > objects contrast elicited a peak activation in the right temporo‐occipital fusiform cortex (26, −44, −16; *Z* = 11.5). The cluster surrounding this peak incorporates parietal regions bilaterally, including precuneus and posterior cingulate cortex, and extends medio‐anteriorly into lingual gyrus. In addition to these posterior activations, significant clusters were found also in right inferior temporal gyrus and middle temporal gyrus bilaterally (see Table 1). By intersecting the whole brain maps with the relevant anatomical ROIs, we found significant bilateral activity in PHG, RSC, TOS, and the HC. The peak voxel in the bilateral RSC ROI was located in the right hemisphere, above the ventral terminus of the parieto‐occipital sulcus (8, −48, 6; *Z* = 7.53). The peak activation in PHG was situated in the posterior division of the structure, within the collateral sulcus (−20, −40, −14; *Z* = 9.38), and the HC peak was located in right anterior HC (22, −16, −22; *Z* = 7.48; anterior/posterior split defined at the uncal apex, see Poppenk et al., 2013). The location of the peak TOS voxel was on the middle occipital gyrus (40, −84, 22; *Z* = 8.19), marginally inferior to the transverse occipital sulcus itself.

**Table 1:** MNI coordinates for the regions identified in the group‐level random‐effects analysis (*n* = 51) (coordinates are displayed for scenes > objects)

*x*	*y*	*z*	*Z score*	*Region*
*Brain regions showing greater activity for scenes > objects*				
26	−44	−16	11.5	Right temporal occipital fusiform cortex
−24	−57	−12	10.3	Left temporal occipital fusiform cortex
20	−60	14	10	Right precuneus cortex
12	−50	0	9.43	Right lingual gyrus
22	−16	−22	7.48	Right hippocampus

#### Percentage signal change in ROIs

Consistent with the regions identified in the whole brain analyses, a significantly greater response for scenes compared with objects was observed in PHG (*t*(1, 50) = 10.26, *P* < 0.01, d = 2.90), TOS (*t*(1, 50) = 7.56, *P* < 0.01, d = 2.14), RSC (*t*(1, 50) = 8.72, *P* < 0.01, d = 2.47), and also the HC (*t*(1, 50) = 6.02, *P* < 0.01, d = 1.70), with a large effect size observed in each ROI (Fig. 3B).

#### Comparison with previous literature

We used NeuroSynth (http://www.neurosynth.org) to assess how closely the locations of our scene‐sensitive group‐level activations (derived from the contrast scenes > objects) correspond to co‐ordinates reported previously in the literature. NeuroSynth is an automated meta‐analytic tool that generates activation maps (based on peak coordinates reported within neuroimaging articles) for certain psychological terms or constructs from a database of 5,809 studies (data retrieved 20/05/14). We used a reverse inference analysis in which the voxel‐wise Z statistic reflects the likelihood that a particular term was used given an activation at that voxel location (a full description of this method can be found in Yarkoni et al., 2011).

The term “scenes” yielded a reverse inference map based on 130 neuroimaging studies with *Z*‐scores ranging from 3.4 to 10.6. This meta‐analytic map revealed activations in posterior PHG bilaterally and, in particular, large *Z*‐scores at the exact location of our left PHG peak (−20, −40, −14, *Z* = 4.03). To determine the number of meta‐analytic voxels located near our activations, we created a 5mm sphere around our group‐defined peak voxels in left and right hemisphere and calculated the number of voxels within that sphere (/81 voxels). In the left hemisphere, there were 26 voxels within 5 mm of our group‐level PHG peak that have been selectively associated with “scenes” in previous studies. In the right hemisphere, we found 22 voxels within 5 mm of the peak for scenes > objects contrast. There were fewer “scenes” voxels near our RSC peak on the meta‐analytic map, and these were solely located in the right hemisphere. Although there was no direct overlap between our peak voxel in the RSC (10, −50, 4; Max *Z* = 4.47) and the “active” meta‐analytic map, there were 7 meta‐analytic voxels within a 5mm radius. For TOS, we likewise identified “active” meta‐analytic voxels within the right hemisphere only. Overall, we identified 47 database voxels within 5 mm of the right group‐level TOS peak.

Consistent with the anterior HC peaks observed at the group‐level, we identified several meta‐analytic voxels within 5 mm of the anterior HC peak in left hemisphere (−22, −14, −20, Max *Z* = 4.96, 8 voxels). There were no meta‐analytic voxels near our right HC peak. In terms of anterior HC more broadly, there were 48 meta‐analytic voxels associated with the term “scenes” located within the anterior HC bilaterally (22, −14, −20; Max Z = 4.63). In summary, these meta‐analyses confirm that, at the group‐level, our peak scene‐selective voxel coordinates correspond well with those reported previously.

### Individual Subject Analysis

#### Percentage signal change

We first compared the proportion of individuals that exhibited a numerically greater BOLD response for scenes compared with objects within each anatomical ROI (each relative to scrambled objects baseline). The majority of subjects were found to have greater activity for scenes over objects in PHG (92% of subjects), RSC (86%), TOS (88%), and HC (78%). From these frequencies we derived a binomial measure that indicated whether scene percent signal change was greater than object percent signal change or not (scored 1 or 0). To test whether the ratio between responses differed across regions (PHG, RSC, TOS, HC) we used a non‐parametric Cochran's Q test. There were no significant differences found between the four anatomical ROIs in the frequency of individuals showing greater activity for scenes over objects (*X* ^2^ (4) = 3.83, *P* = 0.28).

#### ROI analyses

**Figure 4:**
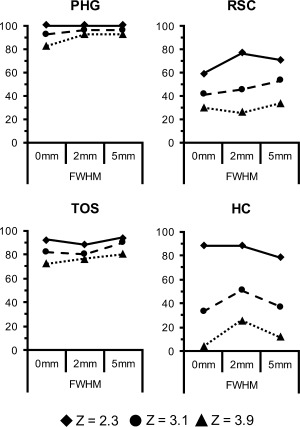
The proportion of individuals with significant clusters in each ROI for the scenes > objects contrast. Data is depicted for each voxel‐wise *Z* threshold (2.3, 3.1, 3.9) and for the three smoothing levels (0, 2, and 5 mm).

Figure 4 displays the proportion of individuals with one or more significant scenes > objects clusters in each ROI. Further, we examined how the proportion of individual‐level activations within each region was affected by (a) the voxel‐wise threshold (*Z* = 2.3; *Z* = 3.1; *Z* = 3.9), and (b) the degree of spatial smoothing applied (unsmoothed (0 mm); FWHM = 2 mm; FWHM = 5 mm).

As can be seen Figure 4, the PHG was found to be highly scene‐selective, independent of threshold and smoothing. At Z = 2.3, 100% of participants had significant clusters in PHG across all spatial filters. Increasing the Z threshold had minimal impact on the proportion of subjects with scene‐selective clusters in PHG, with 92% (47/51) participants possessing significant scenes > objects clusters at *Z* = 3.9. This proportion was slightly higher at FWHMs 2 and 5 mm than with unsmoothed data (82%). Similar to the PHG, TOS was also highly selective, with the majority of participants eliciting individual‐level activation across thresholds and smoothing levels. While individual‐level selectivity was marginally greater with 5 mm smoothing (Fig. 4), more than 70% of participants had significant scenes > objects clusters across all smoothing kernels applied.

In contrast, individual scene‐selectivity in both RSC and HC was greatly affected by the threshold applied. At *Z* = 2.3, a maximum of 76% of participants had supra‐threshold voxels in RSC, which was observed at FWHM = 2 mm. This threshold‐level maximum reduced to 52% at *Z* = 3.1 and 33% at *Z* = 3.9. At lower thresholds, RSC scene‐selectivity was slightly higher at 5 mm smoothing. In HC, there was greater variation in the number of subjects showing scene‐selective activations across different thresholds; at *Z* = 2.3, a striking 88% of subjects elicited scene‐selective activations in HC. Contrary to the other ROIs, this proportion was slightly greater when using the unsmoothed data, or at very low levels of smoothing (2 mm). Reflecting the smaller individual‐level peak *Z* scores, this proportion reduced significantly at *Z* = 3.1, though scene‐selectivity was still comparable to RSC (threshold‐level maximum = 51%). At the most conservative *Z* threshold, scene‐selective voxels in the HC were evident in 26% of participants at FWHM = 2 mm. Overall, HC is the only region where a larger proportion of subjects have suprathreshold voxels at a smaller spatial filter (2 mm), whereas the other ROIs demonstrate a marginal improvement at FWHM = 5 mm.

#### Probabilistic overlap

**Figure 5:**
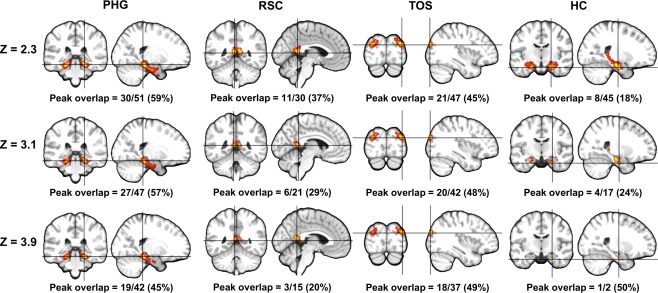
Probabilistic overlap maps of individual‐level activations (unsmoothed) for the scenes > objects contrast at each of the tested cluster thresholds: *Z* = 2.3 (top row); *Z* = 3.1 (middle row); and *Z* = 3.9 (bottom row). The red‐to‐yellow colour scale indicates the proportion of participants with activations in each voxel (yellow = high overlap). The crosshairs indicate the location of the overlap peak on each brain. Both the raw number of participants activating at the peak, and percentage of individual activations (relative to the total number of individual activations for that region and threshold) are reported below each brain image. [Color figure can be viewed at http://wileyonlinelibrary.com.]

To investigate the spatial consistency of activations within each ROI, we created probabilistic overlap maps for each threshold and ROI for the unsmoothed data (see “Materials and Methods” section). To control for differences in the proportion of individual‐level activations across ROIs, percentage statistics are reported relative to the number of individuals with significant activations at that threshold. The highest degree of inter‐subject consistency—as indicated by the voxel with the highest overlap across subjects—was found in the PHG. At all thresholds, this was located in right posterior PHG (24, −34, −16; Fig. 5), and ranged from 59% at *Z* = 2.3 (30/51) to 45% at *Z* = 3.9. Interestingly, this peak is in the opposite hemisphere to that identified in the group‐level analysis, which, when located on the overlap map for *Z* = 2.3 (unsmoothed), constitutes only six subjects. When focusing on the right hemisphere group‐level peak only, this is only 2.8 mm from the overlap peak at all thresholds.

Similarly, almost half (45%) of the subjects tested converged on a single voxel in the right TOS at *Z* = 2.3 (36, −88, 24). Across all thresholds, the peak for the scene contrast was located marginally anterior and lateral to the transverse occipital and intra‐parietal sulci, and situated in the LOC in both hemispheres (Fig. 5, top). This consistent overlap peak (36, −88, 24) was located 6 mm anterior from the group‐level TOS peak for scenes > objects. This high degree of inter‐subject overlap in TOS was also found to increase with increasingly stringent *Z* thresholds (45%–49%) suggesting that the initial peak reflects a convergence of individual‐level maxima, rather than the overlap of cluster edges.

The overlap peak for RSC was consistently located in the left hemisphere, on the medial surface of the posterior cingulate gyrus (12, −52, 8). Relative to the PHG and TOS, above, there was less overlap between individual scene‐selective activations in RSC. At *Z* = 2.3, the overlap peak in RSC reflected an overlap of 37% of individual subject activations; this reduced to 20% at *Z* = 3.9. Furthermore, the individual overlap peak was located in the opposite hemisphere, and anteromedial to, the peak identified at the group‐level.

**Figure 6:**
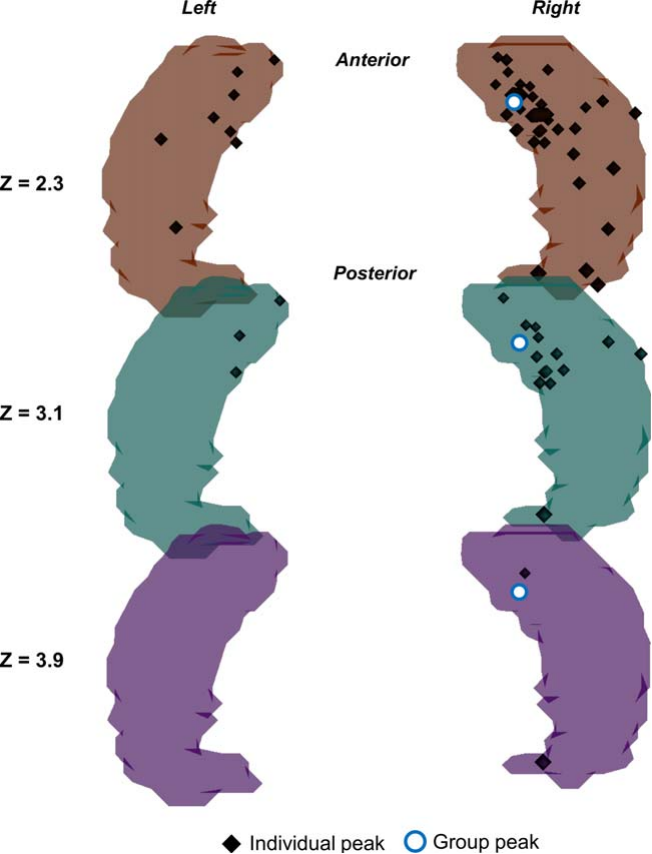
The spatial distribution of individuals' bilateral peak voxels in the HC for scenes > objects. The peak foci are displayed for (**A**) Z = 2.3 (top); (**B**) Z = 3.1 (middle); and (**C**) Z = 3.9 (bottom). The peaks are viewed as if from above, with the anterior at the top and the posterior at the bottom. As these clusters are derived bilaterally, each participant has one peak across both hemispheres. The peaks are illustrated as black markers and the peak overlap voxel for the group analysis is depicted as a blue bordered marker. Coordinates are rendered within the HC anatomical ROI using FSLview. [Color figure can be viewed at http://wileyonlinelibrary.com.]

Across all thresholds, the peak overlap in HC was located in the right anteromedial HC (22, −14, −24), consistent with the group‐level analysis. Despite frequent scene selectivity at *Z* = 2.3 (45/51), there was only 17% overlap in the HC at this threshold, indicating high inter‐individual variability in HC scene activations. Although the proportion of individual‐level activations was found to reduce at higher thresholds (e.g., 17/51 at *Z* = 3.1), the peak overlap nonetheless remains located in anteromedial HC (Fig. 5). To further interrogate the variability of scene responses in the HC, we plotted the peak voxels from each individual (Fig. 6). As can be seen, while the peaks are to some extent distributed across hemispheres and along the long axis, the overlap “peak” converges on the right anteromedial HC (particularly at *Z* = 2.3). At *Z* = 2.3, where most individuals have significant activations (Fig. 6), 73% of individual peak activations are located in anterior HC; this increases further to 82% at *Z* = 3.1. This also corresponds closely with the group‐level results; the peak overlap voxel at *Z* = 2.3 was located 2.8 mm from the group peak. This distance was found to increase at more conservative thresholds to 7.2 mm (as the overlap peak reflects fewer subjects overall).

## GENERAL DISCUSSION

The aim of the current study was to provide a detailed profile of group‐ and individual‐level responses in human scene‐sensitive brain regions. While previous studies have attempted to characterise scene‐sensitivity—and its individual‐level consistency—these studies have often used limited sample sizes [e.g., Nasr et al., 2011; Spiridon et al., 2006], or have restricted analysis to a single brain region [e.g., Peelen and Downing, 2005]. We provide, therefore, the first detailed investigation of scene‐sensitivity within a large sample *and* across a range of regions. While we evaluate the response properties of the “core” scene processing network—namely PHG, RSC, and TOS—a novel aspect of this study was the inclusion of the HC as a component of this network, given its purported role in the representation of complex and conjunctive scenes [Barense et al., 2010; Lee et al., 2008; Mundy et al., 2013] and contributions to scene construction [Maguire and Mullally, 2013; Zeidman et al., 2015].

In addition to demonstrating scene‐sensitivity at the group level (i.e., a greater response to scenes over objects), we also report (a) the proportion of individuals that activate different scene processing regions using an independent anatomical ROI approach, (b) the spatial variability of individual‐level scene responses, and (c) the consistency between individual responses compared with group‐averaged statistics. In this discussion, we summarise the main observations from these analyses before commenting on the theoretical and methodological implications of these results.

### Group‐Level Analyses

Overall, the results of our group‐level analyses were consistent with previous studies that observed strong scene‐sensitivity in these regions during standard localiser tasks [Bettencourt and Xu, 2013; Epstein, 2008; Epstein and Kanwisher, 1998; Lee et al., 2008; Spiridon et al., 2006]. Our main scene‐selective contrast revealed significant bilateral activity in each tested ROI at the whole brain level, including HC and TOS. Comparing the peaks in each ROI with meta‐analytic maps revealed that most group‐level maxima were in the vicinity of scene‐related coordinates reported previously. The group‐level peak for PHG was situated at the most posterior and lateral part of the structure, close to the collateral sulcus and the adjacent temporal fusiform gyrus, as observed previously [Nasr et al., 2011; Park and Chun, 2009]. The RSC peak activation was, again, located near previously reported coordinates (see section “Comparison with Previous Literature”). The group‐level analysis also yielded scene‐related activations in the TOS ROI bilaterally, with the peak voxel in both hemispheres located in the lateral part of the ROI [e.g., Spiridon et al., 2006].

Critically, this one‐back localiser task, which involved rapidly presented scenes with minimal mnemonic demand, yielded strong (*Z* > 7) bilateral activation in the HC bilaterally, supporting a task‐independent role of the HC in complex scene processing [Barense et al., 2010; Lee et al., 2008; Lee and Rudebeck, 2010b]. Unlike several previous studies that have looked at HC responses during scene perception [Barense et al., 2010; Lee et al., 2008; Lee and Rudebeck, 2010b; Mundy et al., 2012; Zeidman et al., 2015], we found stronger group‐level (bilateral) activation in the anterior, rather than posterior, HC (although see Lee et al., 2013). As has been discussed elsewhere, spatial smoothing of EPI data can lead to “blurring” between adjacent anatomical ROIs, such as between PHG and posterior HC [Reber et al., 2002]. Given that our HC peak voxel is located in the anterior HC (∼6 mm from the PHG ROI boundary), blurring of the scene‐selective BOLD response in PHG is unlikely to account for the pattern—and magnitude—of scene activations in the HC.

### Individual‐Level Analyses

At the individual level, there was no difference in the proportion of individuals showing a preferential response to scenes versus objects across the different ROIs. Beyond this, we calculated the proportion of individuals with significant scene‐selective activations within each region. To prevent biasing our results through the selection of arbitrary statistical and pre‐processing criteria, we varied both the voxel‐wise statistical threshold and the smoothing kernel applied. As shown previously [Downing et al., 2006; Epstein et al., 2003], the majority of participants in our sample possessed significant scene‐sensitive clusters in both PHG and TOS, irrespective of threshold and smoothing. In comparison, the RSC showed less individual‐level selectivity, and this was greatly affected by the *Z*‐threshold applied. Overall, while we were able to localise these regions across all smoothing levels, this was marginally better with the larger spatial filter of 5 mm.

As most studies have used anatomical or group‐level ROIs for the HC, rather than clusters defined within individual participants, little was known about individual‐level scene responses in the HC. Here, we demonstrated striking individual‐level selectivity at *Z* = 2.3 in HC, which was (a) comparable with both TOS and PHG, and (b) greater than that shown in the RSC. Similar to the RSC, however, individual HC selectivity was affected by the threshold used, indicating that HC scene activations—while readily identifiable at lower voxel‐wise thresholds—are not as strong as those observed in PHG and TOS. Interestingly, unlike the other ROIs tested, individual‐level selectivity in HC was better using smaller spatial smoothing filters (2 mm). This improvement at lower smoothing, coupled with strong selectivity across all smoothing levels at *Z* = 2.3, is further evidence that these individual‐level HC scene activations are highly unlikely to be the result of signal “bleed” from adjacent PHG.

Probabilistic overlap maps were also generated to determine the spatial consistency of scene activations. In line with its well established role in scene perception and working memory more broadly [Epstein and Kanwisher, 1998; Ranganath et al., 2004], the greatest degree of inter‐subject overlap (across all ROIs and thresholds) was found in posterior PHG (59%). Further, the region of maximum overlap in the PHG was close to the group‐level peak voxel in the same hemisphere. This finding may reflect signal enhancements near the boundary of the PHG ROI, where a large vein overlies the collateral sulcus [Nasr et al., 2011; Turner, 2002].

Consistent with previous work [Spiridon et al., 2006], individual‐level scene activations in TOS were highly consistent. The peak overlap was very close to the group peak and was actually located in the lateral part of the ROI (on the lateral occipital gyrus). The inter‐individual consistency of scene‐selective activations in TOS was somewhat surprising given that the anatomical location of transverse occipital sulcus is highly variable across subjects [Iaria and Petrides, 2007]. This supports the view that the “functional TOS”—or perhaps more appropriately the “occipital place area” [OPA; Dilks et al., 2013]—appears to reside lateral to the sulcus itself, as shown here and in other studies [Nasr et al., 2011; Spiridon et al., 2006]. TOS activations were also found to be more consistent as increasingly stringent thresholds were applied, suggesting that this overlap peak is perhaps more representative of individual‐level peaks when compared with the PHG, which showed a reduction in inter‐subject consistency at more conservative voxel‐wise *Z* thresholds. Individual activations in RSC were more variable than both PHG and TOS. Further, while the right hemisphere group peak was located immediately inferior to the convergence of the parieto‐occipital and calcarine sulcus, the overlap peak was located anterior to this on the medial surface of the right posterior cingulate.

For the HC, both the individual‐subject activation overlap maps and the group‐level analyses point to a consistent peak in anteromedial HC. Relative to the other ROIs tested, however, this peak reflected less inter‐subject overlap. Low peak overlap was observed at *Z* = 2.3, where 45/51 subjects had significant HC clusters. A three‐dimensional (3D) plot of HC peaks at this threshold suggested that while individual activations converged on anteromedial HC, these were somewhat variable along the long axis and across hemispheres.

### Potential Implications

These data have implications for interpreting group‐level maxima/ROIs derived from functional localisers. For example, both the HC probabilistic overlap maps and peak plots suggest that the group‐level peak in anterior HC does not necessarily reflect the average of large and spatially consistent clusters but rather the combination of smaller individual clusters that are potentially highly distributed, both hemispherically and along its long axis. Similarly, the peak voxel in the group analysis for RSC does not reflect the overlap of the majority of participants in the probabilistic map. These findings suggest that when neuroimaging researchers constrain analysis based on a group‐level peak, they may, depending on the specific anatomical structure being studied, overestimate the degree of overlap within a given sample (by assuming the peak reflects high overlap at the individual level) and in some cases reduce the chance of observing an effect by ignoring the peak activation in the majority of tested subjects [Saxe et al., 2006]. Similarly, if a region's response to a particular category, or manipulation, reflects a steep and highly localised peak, then averaging across large anatomical ROIs would result in a reduced and noisier estimate of the response. Our findings, therefore, highlight the importance of using subject‐specific ROIs when extracting individual parameter estimates.

These data also emphasise the importance of using probabilistic atlases—when possible—to characterise the consistency of individual‐level data. As has been discussed previously [Nieto‐Castañón and Fedorenko, 2012], such an approach affords a greater understanding of inter‐individual consistency that is relatively independent of sample size. For example, the wholebrain random‐effects group analysis alone tells us nothing *specific* about inter‐subject consistency, just that a point of overlap exists. A probabilistic atlas based on individually defined activations allows researchers to interpret such peaks in terms of the whole sample (i.e., only 30% of all participants activate voxel x) and from this make probability judgements about how specific peak locations reflect the underlying anatomy (i.e., is there really something scene‐sensitive about posterior HC when only 10% of individuals have scene‐responsive voxels in that area?). In terms of localiser‐based analyses, experimenters can also use these thresholded probabilistic maps to constrain group‐defined ROI analyses, or, when functional localiser data are not available to interrogate an experimental task, use such maps to derive orthogonal ROIs [e.g., Engell and McCarthy, 2013].

### Comparing Regions

As discussed above in section “Individual‐Level Analyses,” there were *inter‐region* differences in terms of both frequency (the proportion of subjects possessing significant clusters) and consistency (the extent to which subjects activate the same voxels). These need not, however, reflect differences in the degree of scene‐sensitivity *per se*. As discussed by other authors [Duncan et al., 2009; Rossion et al., 2012; Todorov, 2012], it is difficult to determine *a priori* the appropriate statistical thresholds for detecting activations within a given brain region. The reason for this may arise from several factors; for instance, the shape (and height) of the HRF has been shown to vary across both individuals and brain regions [Aguirre et al., 1998b; Goense et al., 2012; Handwerker et al., 2004]. Moreover, as MTL regions (such as HC) are located nearer the air/tissue interfaces, they are highly susceptible to magnetic field distortion and reduced signal‐to‐noise [Olman et al., 2009]. It is notable, therefore, that the *Z* threshold applied in our analyses had the most striking impact on the frequency and consistency of activations in HC (Fig. 4). Critically, then, reduced signal‐to‐noise could be one factor underpinning differences in inter‐individual consistency between the “core” regions and the HC.

While a “core” network of regions has been shown consistently to respond more to scene stimuli than other visual categories, as indicated by the meta‐analytic analysis above, important functional differences between regions have been identified. Overall, posterior PHG appears to have a more general role in scene processing, possibly reflecting a viewpoint‐independent processing of scene structure [Epstein et al., 2007; Epstein and Higgins, 2007] that is insensitive to scene familiarity [Epstein et al., 1999; Epstein, 2008] and the position of a scene within its broader environment [Epstein et al., 2003; Epstein, 2005; Park and Chun, 2009]. This conclusion is supported by evidence that posterior PHG is sensitive to spatial boundary [Park et al., 2011], rectilinearity [Nasr et al., 2014], and motion within visual scenes [Korkmaz Hacialihafiz and Bartels, 2015]. PHG also shows a greater response to scenes with low, compared with high, feature overlap [Mundy et al., 2012]. Electrophysiological studies in humans also indicate that cells in this region show greater response when viewing spatial landmarks, whereas HC neurons respond to specific locations within an environment [Ekstrom et al., 2003]. Thus, posterior PHG may support navigation via its role in the visual processing of scenes/landmarks [see also Janzen and van Turennout, 2004], rather than coding allocentric position within space [Ekstrom, 2015; Hartley et al., 2003]. To this extent, therefore, functional localiser tasks (like the one used here), which often involve the presentation of unfamiliar and featurally non‐overlapping spatial scenes/landmarks could be argued to place demand on these visually driven, viewpoint‐dependent scene representations in posterior PHG [Ekstrom, 2015; Epstein et al., 1999; Mundy et al., 2012; Park and Chun, 2009].

Like posterior PHG, TOS scene activations were both readily identifiable and highly consistent across subjects. The emerging view from recent neuroimaging studies is that the TOS, while playing an important and causal role in scene perception [Dilks et al., 2013], may support the processing of lower‐level spatial features [Nasr et al., 2014]. As such, studies have found a greater response in TOS when performing spatial judgements about dot stimuli [Nasr et al., 2013], when viewing scene motion [Korkmaz Hacialihafiz and Bartels, 2015], and when viewing rectilinear features [Nasr et al., 2014]. The latter study, in particular, found that the difference between cubes and spheres in the TOS was greater than scenes versus faces, thus underlining the TOS as a potential lower‐level scene‐processing region. While we observed a large response to scenes relative to objects in this study, we are unable to say whether this reflects higher‐level scene representations in TOS, such as scene category recognition or spatial layout [Dilks et al., 2013], or inherent differences in lower‐level stimulus attributes [see also Bryan et al., 2016].

In terms of RSC, both the animal and human literature indicates that RSC is an important brain region for spatial navigation [Byrne et al., 2007; Epstein and Vass, 2014; Knight and Hayman, 2014; Kravitz et al., 2011b; Vann et al., 2009]. As such, RSC responses have been shown to attenuate across multiple views of the same scene, suggesting that this region maintains information about the local environment across visual transformations [Marchette et al., 2014; Park and Chun, 2009]. This region also seems sensitive to landmark information by showing parametric increases in response to landmark “permanence” [Auger et al., 2012] and the size of visual scenes [Park et al., 2015], thus reinforcing the view that the RSC codes information that is relevant to navigation, such as allocentric spatial layout. As stated above, we found less consistency in RSC relative to PHG and TOS, and there was less correspondence between group‐level RSC peaks and those derived from the probabilistic overlap. This was reflected also in the meta‐analytic maps, in which few voxels were located in the regions of RSC and posteromedial cortex, reflecting a lack of consensus across studies. The indication from previous literature, therefore, is that scene clusters in RSC are somewhat variable, independent of the anatomical protocol used in a given study (Brodmann areas, manual segmentation, etc). Also, while the one‐back localiser task was sufficient to identify significant RSC voxels in individual brains, particularly at lower voxel‐wise thresholds, it is not necessarily attuned to the precise function of this brain region in scene processing (i.e., spatial navigation), which could account for the reduced inter‐individual consistency found here. In addition, there are inconsistencies in the field when defining the RSC [Knight and Hayman, 2014; Vann et al., 2009], to the extent that some researchers have restricted analysis to anatomical boundaries [Auger and Maguire, 2013], whereas others use the more liberal, and functionally‐defined retrosplenial *complex* [Bar and Aminoff, 2003; Epstein et al., 2007].

An important aspect of the current study was including the HC as one of the key scene‐sensitive ROIs. Although inter‐individual consistency was low in our analyses, the proportion of individual‐level activations in HC (coupled with robust group‐level activations) was shown to be comparable with “core” scene‐sensitive regions, and provides further support for the role of the HC in scene processing [Bird and Burgess, 2008; Graham et al., 2010; Hassabis and Maguire, 2009]. While studies in rodents highlight the HC as critical for spatial navigation and allocentric spatial processing, as evidenced by the presence of place‐selective cells in HC [O'Keefe and Nadel, 1978] and spatial memory deficits following HC lesions [Morris, 1984], the notion that the human HC is involved in scene processing remains controversial [Suzuki, 2009]. While the human HC may be important for navigation [Ekstrom et al., 2003; Maguire et al., 2000; Suthana et al., 2009], its recruitment across a range of mnemonic and perceptual scene processing tasks is indicative of a broader role in scene cognition. Here, too, we localised HC using a working memory task, thus complementing previous studies that find evidence for HC recruitment across a range of cognitive tasks. Indeed, several studies of patients with HC atrophy have reported scene‐specific deficits for both memory and perceptual tasks [Bird et al., 2008; Hartley et al., 2007; Lee et al., 2005a, 2005b; Mullally et al., 2012; but see Kim et al., 2011, 2015]. Functional neuroimaging studies have likewise found group‐level HC activation during scene discrimination [Aly et al., 2013; Lee et al., 2008], scene construction/imagining [Zeidman et al., 2015], and working memory [Lee and Rudebeck, 2010b; Park et al., 2003]. Studies applying multivariate analysis techniques have also found evidence that the HC contains activation patterns that are sensitive to scene‐related information [Bonnici et al., 2012; Liang et al., 2013; but see Diana et al., 2008]. Overall, these extant data, when viewed alongside the large‐scale analysis of individual‐level data reported here, suggest that the HC should be considered a key region in the putative scene processing network [see also Kornblith et al., 2013; Kreiman et al., 2000]. While these results implicate HC in the online processing of visual scenes—contrary to the perspective that HC activation is primarily driven by internally generated thought [Buckner et al., 2008]—it is not possible in this block design study to determine whether HC response is being driven by perceptual processing or scene maintenance across trials [Zeidman et al., 2015].

A role for the HC in scene processing is further evidenced by its strong anatomical connectivity with other regions in this “core” scene network [Kravitz et al., 2011b]. Studies in animals have shown, for example, that a parietal‐medial temporal pathway linking the posteromedial cortex (including RSC), posterior PHG and the subiculum/CA1 of the HC may be a key pathway in the processing and transfer of complex visuospatial information [Kravitz et al., 2011b]. Drawing correspondence with the findings reported here, the anteromedial subiculum, in particular, has been highlighted as a potential key sub‐region in human scene processing [Aggleton, 2012; Zeidman et al., 2015]. While we do not have the necessary resolution to explore subfield contributions to scenes here, our findings are nonetheless consistent with a role of the HC in scene processing, as underpinned by its position within a broader network for spatial navigation and scene processing [Aggleton, 2012].

## CONCLUSIONS

In the current study we conducted a detailed analysis of both group‐ and individual‐level responses in several brain regions that are considered to show preferential responses to scene or place stimuli. While focussing on the putative scene processing network namely PHG, RSC, and TOS, we make a further novel contribution to the literature by evaluating also the response profile of the HC, which has recently been identified as critical for successful higher‐order scene perception [Lee et al., 2012]. By concatenating functional localiser data across multiple studies, we were able to characterise—within a large sample—the frequency and spatial consistency of individual scene‐sensitive activations in these regions. Overall, we demonstrated preferential scene responses in both the “core” scene‐processing network and the HC, and these were detectable at the group, and more interestingly, at the individual level. Both analyses potentially highlighted a key role of the anteromedial HC in scene processing. Importantly, this study provides a framework for evaluating individual‐level responses that could be applied to other visual categories and/or experimental tasks, and highlights that certain approaches (e.g., frequency of activation, probabilistic overlap) can provide important insights that are not only theoretically informative but have implications for how functional data are interpreted in the context of visual cognition and category‐selectivity.

## Footnotes

1 The term “anatomical mask” is adopted throughout to reflect the fact that all masks are based broadly on anatomical properties, albeit derived through different methods. As is discussed in the methods, the HC, TOS, and PHG masks are based on probabilistic atlases (where individual‐level segmentations are overlaid within a standard atlas space) and the RSC is based on cytoarchitectonic boundaries defined by Brodmann, that is, BA29.
